# Modus Operandi of Anæsthetics

**Published:** 1869-09

**Authors:** 


### Modus Operandi of Anæsthetics.

In a letter to Dr. J. H. McQuillen, of the *Dental Cosmos*, Dr. A. E. Sansom, Physician to Kings Coll. Hospital of London, gives the following statement deduced from his researches on this subject:

A. We have no evidence that in anæsthesia there is any direct union between the anæsthetic and nerve matter. Evidence tends rather to the contrary conclusion.

B. We have no evidence that in anæsthesia there is (as some have held) any impaired *oxidation of nerve matter.* We have no evidence whatever that the normal functions of nerve are due to any oxidation of its substance, much less that the absence of these functions are due to the impairment of oxidation.

C. We have positive data to this effect: that the suspension of supply of arterial blood can impair nerve function, and that impairment of the quality of the blood (suspension of oxidation) will impair nerve function.

D. We find that anæsthetics cause contraction of the channels of arterial blood-supply. Conversely (as in narcotism from cold,) where there is adequate contraction of arterial vessels, there is narcotism.

E. We find that anæsthetics added to the blood impair its powers of æration (see Harley's Experiments.) How do they effect this impairment? The only positive data on which we can base any answer are—(1) the fact that anæsthetics do, when added to the blood, impair its form and integrity; we can argue, a fortiori, from the observed effects of the more concentrated influences to the possible effects of the feebler ones; (2,) or, that these agents have a chemical action on the proteinous matter of the blood corpuscle.

I contend that in C and E we have a sufficient explanation of the phenomena of narcosis.

